# The State and the Shop

**Published:** 1892-08-27

**Authors:** 


### The Hospital

An Institutional Journal of

Science, Medicine, Nursing, and Philanthropy.

*For Hospitals, Asylums, Infirmaries, Dispensaries, Medical Practitioners, Students, Nurses, and the Charitable Public.*

Vol. XII.—No. 309.] [Aug. 27, 1892.

### THE STATE AND THE SHOP.

It seems but yesterday that a French king to whom an appeal was made for a change of decision on the ground of considerations of State, replied, "The State! I am the State." Monarchs are wiser now, happily for their subjects and for themselves. Shop assistants constitute a numerically large and socially important part of the State. They belong to the self-respecting and fairly-educated classes. They have for the most part a reasonable propect of obtaining a "stake in the country." The drift of their mind is towards stable political institutions. Anarchy is as dangerous to their interests and prospects as it is to those of the wealthier classes. By descent, by training, by instinct, and by mental culture they are inclined to range themselves on the side of industry, respectability, and the accumulation and preservation of property.

If this be a just diagnosis it is evident that such a class is worthy of serious consideration in the present condition of British politics. The physician sees an aspect of politics which does not strike the ordinary statesman. He sees, for example, the causes that produce irritable brains and hypersensitive nervous systems. He knows, and every thinking person knows, that hyper-sensitive nervous systems are very combustible materials. What man who has a wife and children and servants has not seen storms brewing and bursting on the domestic hearth which had their origin, not in the electrical condition of the household affairs, but in the dim recesses of one or more over-sensitive nervous systems? As the peace of the home often depends upon the amount of sobriety and self control residing in the brain of each member of the household, so do the steadiness and strength of the State depend upon the sober-minded and capable industrialism of the self-respecting toilers.

Sound health is the parent of satisfied citizenship: feeble health is the progenitor of discontent and all manner of unreasonableness. The physican does not treat discontent and unreasonableness originating in bad health by scoldings, repressions, and coercions; he sets to work to put the health right. When that is done the discontent and unreasonableness cure themselves. Now, the shop assistants of this country live and work under such conditions that health is impossible. Their average hours are not less than thirteen a day. If their work were out of doors the number of hours would not greatly matter from a health point of view. But it is indoors, all indoors; and their atmosphere is poisoned during the hours of daylight by the manifold breath of customers and assistants, and during the hours of the evening by the jets of gas which blaze on every hand. It has been pointed out times without number that such long hours in such atmospheres are ruin and destruction to the young women who constitute the larger number of shop assistants. They first become bloodless, then fleshless, then spiritless, and then worthless. They do not, it is true, set up standards of rebellion, or strive by force to emancipate themselves from a condition which is slavery without its ameliorations. But they die, or, what is worse, marry and become mothers of children as bloodless, as spiritless and as useless as themselves.

But all these evils are not inflicted upon the young women alone. The young men are subject to exactly the same disabling and health destroying conditions. They have a little more stamina than the women, and so they break down more slowly; but if we could see a regiment of London shop assistants of thirty years old and upwards, they would be a veiy sorry spectacle of British manhood. The artisans and mechanics of our country have far greater possibilities of health and well-being than the shop assistants. Not only have they shorter hours of work, but their duty takes them more out doors, and they have longer intervals for meals; but in addition, the physical exercise which their work gives them is of inestimable advantage. Of this advantage shop assistants are almost entirely deprived.

The writer was struck the other morning with the extraordinary quantity of matter in his daily newspaper which was devoted exclusively to the affairs of the artisan class. Not less than sixteen or seventeen columns of one paper were entirely given up to these affairs. It is evident, indeed, to all of us that the artisans are prepared to take very good care of themselves. Nay, it is manifest that they will in no long time have a good deal of superflous strength. It would be no unnatural thing if shop assistants, who do not now belong to the artisan class, and who cling desperately to their own somewhat superior order, should, in despair of sympathy and help from any other quarter, go over to trades unionism and all the methods of the social revolutionary. Who could blame them if they were to do so? Human nature has no more than a certain power of endurance, and when a superior class sees an inferior getting a long way ahead of it in the life struggle, what wonder if it should cast longing eyes at the methods which have brought about so enviable a result. It would be something of a calamity if the shop keeping element were driven perforce into discontent and ıevolution.

The more stable classes of society, if they were wise, would take steps to attract allies to their own standard. Liberals as well as Conservatives feel already the surges that are heaving in the profound social depths; and Liberals as well as Conservatives are becoming clearly aware that the forces of order and stability will soon need every available augmentation if the good ship of the State is to ride securely on the rising flood.

The morning paper which devoted sixteen of its columns to the artisan class spared about a sixth of one column for the recording of a few significant facts about shop assistants. It seems that the Early Closing Association have been successful in securing a weekly half-holiday in the metropolitan districts of Holloway, Hackney, Richmond, Ealing, and Hounslow. This is a very important step in the right direction, and, we may hope, the precursor of a good many more. If Parliament is to take in hand the question of the hours of miners and other less skilled labourers, much more should it take in hand the question of the hours of shop assistants. The miners can take care of themselves, and so can the artisans generally. The shop assistants cannot take care of themselves: all the conditions of our social life fight against them. All classes shop late in the day; the working classes latest of all. It is obvious that shopkeepers cannot be expected voluntarily to close their places of business during the very hours when the public are most abroad. Of course, if they could all agree to do this, and could depend upon every member of their class to stand loyally by their agreement there would be no difficulty. But that is impossible so long as human nature is what it is. There are always some who will play the part of the greedy knave, even if their whole class be reduced to ill health and misery thereby.

The closing of retail shops at a definite hour by State or municipal authority would be a measure which might be adopted without injury to anybody. The shopkeepers themselves would undoubtedly welcome such a measure if it were made compulsory upon all their class. It is true that the working classes do their shopping principally in the evening, but that is no reason why every evening in the week should be devoted to their selfish tardiness. Two evenings in each week, say Wednesday and Saturday, ought to be ample for such extra shopping as the working class could not do in the day-time. All the other evenings of the week ought to be given up to the health and well-being of the shop assistant. Shops ought to be closed and all business brought to a termination not later than seven o'clock in the evening, but earlier if possible.

To ask for sympathy and pity for the shop assistant is not our present purpose. We ask the State, which is the public, to consider whether there is not in this question a means of strengthening those forces that make for stability, justice, and the maintenance of every man's right to the fruits of his own industry. There can be no greater folly than first to drive men into the madness of revolution and then to control them by means of the straight waistcoat. Cure the disease and the symptoms will disappear. Make life worth living for the law-abiding classes and they in turn will be the sturdiest bulwarks of law and all that is conserved by law. A "Nation of Shopkeepers" should preserve the life of the shop.

